# Investigating oral health among US adults with sleep disorder: a cross-sectional study

**DOI:** 10.1186/s12903-023-03686-5

**Published:** 2023-12-13

**Authors:** Emad Movahed, Shayan Moradi, Bardia Mortezagholi, Arman Shafiee, Hassan Moltazemi, Hamed Hajishah, Sepehr Siahvoshi, Ayad Bahadori Monfared, Mohammad Javad Amini, Farima Safari, Mahmood Bakhtiyari

**Affiliations:** 1 School of Dentistry, RWTH Aachen University, Aachen, Germany; 2 School of Medicine, Tehran University of Medical Sciences, Tehran, Iran; 3 Dental Materials Research Center, School of Dentistry, Islamic Azad University of Medical Sciences, Tehran, Iran; 4 Non-communicable Diseases Research Center, Alborz University of Medical Sciences, Karaj, Iran; 5 Student Research Committee, School of Medicine, Alborz University of Medical Sciences, Karaj, Iran; 6 Alborz University of Medical Sciences, Hassan Abad, Karaj, Alborz Province Iran; 7 School of Medicine, Islamic Azad University of Medical Sciences, Tehran, Iran; 8 Department of Health and Community Medicine, School of Medicine, Shahid Beheshti University of Medical Sciences, Tehran, Iran; 9 Student Research Committee, School of Medicine, Shiraz University of Medical Sciences, Shiraz, Iran

**Keywords:** Sleep wake disorders, Oral health

## Abstract

**Background:**

This study aims to investigate the relationship between sleep disorders and oral health outcomes among a representative sample of the United States population.

**Methods:**

The study sample comprised 6,161 participants who participated in the NHANES 2017–2018, representing a population of 255,939,599. Oral health outcomes were assessed using the Oral Health Questionnaire (OHQ), covering dental pain, periodontal disease, bone loss, emotional perceptions of oral health, and impact on daily life. Sleep disorders were evaluated using questions related to sleep trouble and daytime sleepiness.

**Results:**

Analysis of the NHANES 2017–2018 dataset, revealed notable associations between sleep disorders and oral health outcomes. Individuals with sleep disorders were more likely to report dental pain (19.79% vs. 11.8%), periodontal issues (19.5% vs. 12.25%), and feeling bad or embarrassed about their oral health (21% vs. 12%), compared to those without sleep disorders. Difficulty due to oral health issues was also more prevalent among participants with sleep disorders (32.6% vs. 12.9%). Adjusted models demonstrated that individuals with sleep disorders had a significantly higher likelihood of experiencing oral aches [adjusted odds ratio (aOR) = 1.58 (1.22–2.22)], reporting negative emotions about oral health [aOR = 1.59 (1.06–2.37)], and encountering challenges in school or job performance [aOR = 2.27 (1.47–3.51)], compared to individuals without sleep disorders (refer to Table 3). Other significant covariates affecting oral health outcomes included smoking, income, and education level.

**Conclusions:**

This study reveals a compelling association between sleep disorders and adverse oral health outcomes in the U.S. population.

## Introduction

A crucial element of overall well-being and quality of life is oral health. It is essential for enabling one to comfortably eat, communicate, and interact with others as well as for affecting one’s confidence and self-worth [1]. The maintenance of good dental health depends on a number of variables, such as oral hygiene routines, food patterns, and lifestyle decisions. However, a growing body of research points to a potential link between sleep disturbances and dental health, two areas of health that have recently drawn a lot of attention [2].

Sleep disorders, such as insomnia, sleep apnea, and restless leg syndrome, are prevalent conditions that can disrupt an individual’s sleep patterns and, consequently, their overall health. These disorders often result in insufficient or poor-quality sleep, leading to various physical and mental health issues. While previous studies have extensively explored the adverse effects of sleep disorders on systemic health, limited research has investigated their potential implications for oral health [3–6].

The prevalence of sleep disorders has been on the rise in the United States, with millions of adults experiencing symptoms that affect their sleep quality and duration [7]. Simultaneously, oral health issues, including dental caries, periodontal diseases, and tooth loss, continue to be prevalent and affect individuals of all age groups [8]. Given the potential interplay between these two health domains, it is imperative to investigate whether sleep disorders are associated with a higher risk of oral health problems. Therefore, in this study, we aim to assess the relationship between sleep disorders and oral health outcomes from 2017 to 2018 using the National Health and Nutrition Examination Survey (NHANES).

## Methods

### Study population

This study aims to investigate oral health among individuals with sleep disorders using data from the National Health and Nutrition Examination Survey (NHANES) conducted in 2017–2018. The NHANES is a cross-sectional survey that collects comprehensive health and nutrition data from a representative sample of the United States population. The NHANES protocol, including data collection and definitions, was approved by the National Center for Health Statistics (NCHS) Research Ethics Review Board. Participants provided informed consent, and compensation was provided alongside a report of medical findings for their participation in the survey. The study sample consisted of adults who participated in the NHANES 2017–2018 assessment.

### Data collection

The NHANES 2017–2018 assessment included various examination components, such as medical, dental, and physiological measurements, as well as laboratory tests. These examinations were conducted by trained medical personnel using modern equipment to ensure reliable and high-quality data collection.

### Variables definition

To evaluate oral health outcomes, we used the Oral Health Questionnaire (OHQ). These inquiries covered details about their oral health, with ratings ranging from Excellent to Very Good, Good, Fair, and Poor. We also looked at other factors that affected oral and dental health, like bone loss, periodontal disease, and the existence of tooth pain. We also recorded the participant’s quality of life markers, such as whether they ever felt bad emotions or embarrassed about their oral health and whether they missed work or had problems at their employment as a result of tooth pain.

For assessing sleep disorders, we used a combination of two questions to develop a sleep disorder module based on the available data in NHAES dataset (“Ever told doctor had trouble sleeping?” and “How often feel overly sleepy during day?”). The duration of sleep was then divided into three categories: less than 7 h, between 7 and 9 h, and greater than 9 h [9]; 2) For the second variable, we included the question “Ever told doctor had trouble sleeping?” and defined it as a sleep trouble variable.

The selection of covariates was guided by relevant logic and previously published literature. Using standardized questionnaires, we gathered demographic data on subjects’ age, gender, racial and ethnic background, level of education, marital status, annual family income, drinking and smoking habits, and history of medical conditions (including diabetes, asthma, and coronary heart disease). Non-Hispanic white, non-Hispanic black, non-Hispanic Asian, Hispanic, and other racial/ethnic groupings were established. Education was divided into two categories: a diploma or above and less than a high school diploma. There were two categories for marital status: married/having a, or single. Based on their answers to questions about whether or not they had ever smoked at least 100 cigarettes in their lifetime and whether or not they currently smoked, smoking history was separated into past and present smokers. Based on the response to the inquiry, “Have you consumed at least 12 alcoholic drinks in a year?“ alcohol consumption status was calculated. Based on replies to the medical condition questionnaire, past medical conditions were detected, with each disease requiring a doctor’s prior diagnosis.

### Analysis

We conducted data analyses using StataCorp. 2017. Stata Statistical Software: Release 15. College Station, TX: StataCorp LLC. To ensure the representativeness of our findings, we applied a two-year sample weight to all the data. In order to compare the sociodemographic characteristics, covariates, and oral health outcomes of individuals with and without sleep disorder, we carried out bivariate analyses. For continuous variables like age, we employed the Wilcoxon rank-sum test, while categorical variables were analyzed using the chi-square test, and weighted percentages were reported. To account for the complex survey design, we utilized the Full sample two-year MEC exam weight, the Masked variance pseudo-PSU for clustering, and the Masked variance pseudo-stratum for stratification. We presented weighted frequencies, adjusted odds ratios, 95% confidence intervals (CIs), and p-values. Statistically significant results were identified with a p-value of less than 0.05. To investigate the relationship between sleep disorders and oral health outcomes, we conducted logistic regression analyses.

## Results

The association between sleep disorder and oral health related outcomes was evaluated by analyzing data from the NHANES 2017–2018 dataset. A total of 6,161 participants, representing a population of 255,939,599, were examined. Table 1 presents the demographic characteristics of the population involved in the study.

**Table 1:** Characteristics of included participants

Participant Characteristics	Independent variable					*P* value
	Total	Without Sleep disorder		With sleep disorder		
	N	N	%	N	%	
**Gender**						
Male	2991	2289	35.79	202	12.50	0.1097
Female	3165	2246	35.06	919	16.64	
**Race/Ethnicity**						
White	2123	1782	85.25	341	14.75	0.0010
Black	1415	1281	90.71	134	9.29	
Mexican American(?)	2623	2422	90.51	201	9.49	
**Education**						
Less than 12 years	1117	1007	89.38	110	10.62	0.0412
More than 12 years	4439	3919	86.63	520	13.37	
**Marital Status**						
Married/Partner	3252	2926	87.73	326	12.27	0.0408
Single	2311	2007	85.69	304	14.31	
**Income**						
Below Poverty	984	850	83.80	134	16.20	0.0919
Above Poverty	4256	3797	87.29	459	12.71	
**Alcohol**						
Yes	4545	3986	86.31	559	13.69	0.0453
No	585	548	91.99	37	8.01	
**Smoker**						
Yes	2359	2020	83.64	339	16.36	0.0011
No	3497	3179	89.47	318	10.53	
**Diabetes**						
Yes	883	729	78.04	154	21.96	0.0000
No	5095	4591	88.46	504	11.54	
**Sleep Pattern**						
Short	1496	1300	84.27	196	15.73	0.1018
Normal	3118	2817	88.50	301	11.50	
Long	1499	1332	87.81	167	12.19	

Table 2 presents the relation between symptoms of sleep disorder and various oral health outcomes. Regarding dental pain, 19.79% of individuals experiencing sleep disorder reported dental discomfort, while only 11.8% of those without sleep disorder did. In the case of periodontal disease, 19.5% of individuals with sleep disorder had periodontal issues, compared to 12.25% of those without sleep disorder. As for bone loss, 14% occurred in the sleep disorder group, while it was 13.6% in the non-sleep disorder group. Participants experiencing sleep disorder reported feeling bad or embarrassed about their oral health at a rate of 21%, whereas only 12% of the non-sleep disorder group felt the same way. Difficulty due to oral health issues was reported by 32.6% of individuals with sleep disorder and by 12.9% of those without sleep disorder.

**Table 2:** Association between oral health outcomes of persons with and without sleep disorder

Oral health outcome	Independent variable					*P *value
	Total	Without Sleep disorder		With sleep disorder		
	N	N	%	N	%	
**Had aching in the mouth**						
No	3492	3164	88.2	328	11.8	0.0003
Yes	1246	1020	80.21	226	19.79	
**Had difficulty with job/school**						
No	4472	3987	87.1	485	12.9	< 0.001
Yes	268	199	67.4	69	32.6	
**Felt bad/embarrassed**						
No	3741	3368	88	373	12	0.0015
Yes	998	817	79	181	21	
**Periodontal disease**						
No	3784	3374	87.75	410	12.25	0.0006
Yes	894	757	80.5	137	19.5	
**Condition of teeth and gum**						
Poor	1963	1678	83	285	17	0.0007
Good	4191	3801	88.9	390	11.1	
**Teeth bone loss**						
No	3908	3469	86.4	439	13.6	0.8619
Yes	792	685	86	107	14	

In the adjusted models, individuals experiencing sleep disorder exhibited a higher likelihood of poor oral health outcomes, including those experiencing oral aches [aOR = 1.58 (1.22–2.22)] in comparison to those without sleep disorder (*p* = 0.012). Additionally, individuals with sleep disorder were more prone to feeling negatively about their oral health and experiencing embarrassment [aOR = 1.59 (1.06–2.37)], as well as encountering challenges in their school or job performance [aOR = 2.27 (1.47–3.51)] when contrasted with individuals not experiencing sleep disorder. Furthermore, smoking, income, and education level were significant covariates affecting oral health outcomes of participants (refer to Table 3).

**Table 3:** Multivariate analysis for sleep disturbance and covariate indicators by preventive measures and clinical presentation of oral health outcome

	Had aching in the mouth			Had difficulty with job/school			Felt bad/embarrassed			Periodontal disease			Condition of teeth and gum			Teeth bone loss		
	OR	95%CI	*P* value	OR	95%CI	*P* value	OR	95%CI	*P* value	OR	95%CI	*P* value	OR	95%CI	*P* value	OR	95%CI	*P* value
**Sleep disorder**																		
No	Ref			Ref			Ref			Ref			Ref			Ref		
Yes	1.58	1.22–2.22	**0.012**	2.27	1.47–3.51	**0.001**	1.59	1.06–2.37	**0.025**	1.60	1.12–2.29	**0.013**	0.77	0.57–1.03	0.077	0.91	0.54–1.54	0.728
**Sleep pattern**																		
Short	Ref			Ref			Ref			Ref			Ref			Ref		
Normal	0.66	0.52–0.83	0.002	0.44	0.27–0.72	0.003	0.64	0.43–0.96	0.034	0.73	0.54–0.99	0.046	1.52	1.28–1.81	0.000	0.89	0.57–1.38	0.585
Long	0.81	0.56–1.18	0.261	0.70	0.42–1.15	0.148	0.77	0.54–1.08	0.131	0.97	0.65–1.45	0.885	1.20	0.96–1.52	0.099	0.92	0.52–1.62	0.767
**Race**																		
White	Ref			Ref			Ref			Ref			Ref			Ref		
Black	1.34	0.98–1.44	0.062	1.83	0.88–3.80	0.094	1.05	0.81–1.36	0.679	0.81	0.54–1.19	0.269	0.61	0.45–0.83	0.004	0.92	0.64–1.33	0.667
Mexican American	1.16	0.84–1.61	0.329	1.96	1.13–3.40	0.020	1.02	0.77–1.34	0.854	0.86	0.64–1.16	0.309	0.74	0.56–0.96	0.028	1.11	0.80–1.53	0.483
**Education**																		
Less than 12 years	Ref			Ref			Ref			Ref			Ref			Ref		
More than 12 years	0.76	0.58-1.00	0.057	0.47	0.33–0.69	0.001	0.65	0.48–0.87	0.008	0.80	0.61–1.04	0.092	2.52	2.05–3.10	0.000	1.01	0.74–1.38	0.924
**Marital status**																		
Single	Ref			Ref			Ref			Ref			Ref			Ref		
Married/Partner	0.87	0.69–1.10	0.247	0.71	0.52–0.97	0.036	0.65	0.48–0.88	0.009	1.04	0.75–1.45	0.777	1.02	0.82–1.27	0.827	0.86	0.61–1.21	0.391
**Income**																		
Below Poverty	Ref			Ref			Ref			Ref			Ref			Ref		
Above Poverty	0.62	0.49–0.79	0.001	0.24	0.15–0.39	0.000	0.41	0.32–0.52	0.000	0.67	0.47–0.94	0.025	2.30	1.83–2.90	0.000	0.97	0.66–1.41	0.884
**Alcohol**																		
No	Ref			Ref			Ref			Ref			Ref			Ref		
Yes	0.81	0.39–1.68	0.557	1.97	0.97-4.00	0.059	1.37	0.69–2.70	0.338	0.61	0.31–1.20	0.144	0.85	0.58–1.24	0.390	1.73	1.02–2.90	0.040
**Smoking**																		
No	Ref			Ref			Ref			Ref			Ref			Ref		
Yes	1.58	1.27–1.97	0.000	2.14	1.26–3.63	0.008	2.16	1.67–2.79	0.000	1.62	1.23–2.13	0.002	0.47	0.40–0.54	0.000	2.00	1.57–2.56	0.000
**Diabetes**																		
No	Ref			Ref			Ref			Ref			Ref			Ref		
Yes	1.18	0.77–1.80	0.400	1.49	0.91–2.43	0.098	1.38	1.06–1.81	0.020	1.19	0.81–1.73	0.336	0.62	0.40–0.97	0.038	1.05	0.60–1.85	0.838

## Discussion

We were privileged to utilize the National Health and Nutrition Examination Survey (NHANES) in our study. This survey granted us access to two critical components: a well-established sleep disorders screening tool, the SLQ [10], and comprehensive oral health information. Our research suggests an association between sleep disorders, oral health complications (as related to quality of life), and dental pain. Our investigation further implies that individuals with sleep disorders may encounter emotional challenges associated with oral health problems. However, we found no association between sleep disorders and bone loss, potentially due to individuals’ unawareness of their alveolar bone issue. It may also be that these outcomes are difficult to diagnose without healthcare professionals’ involvement. Specialized equipment, such as X-rays and periodontal probes, are necessary for detecting subtle changes in bone loss, yet the link between sleep disorders and bone loss is of practical significance.

Numerous studies outline the relationship between sleep disorders and dental health [11–18]. However, the limitations of these studies include the inclusion of children of 17 years and younger as participants [16] and the analysis of single oral health outcomes such as xerostomia [19], bruxism [20], and dental caries [21]. Acar et al. [22] identified a significant positive association between the duration of snoring complaints and dental and oral health reports, along with a negative correlation between income levels and educational status. No relationship was found connecting the DMFT index and the severity of obstructive sleep apnoea syndrome. A strong association was found between obstructive sleep apnea in children and deteriorated oral health [16]. Furthermore, Han et al.‘s [15] study uncovered a significant relation between sleep duration in individuals under the age of 60 and their oral health status. There is evidence suggesting that patients with sleep disorders, such as obstructive sleep apnea and snoring, have a reduced masticatory function due to fewer teeth [23]. In accordance with other studies, tooth loss is indeed an oral issue that may be related to sleep disorders like obstructive sleep apnea and snoring [24, 25]. This reduction has been proposed to be a consequence of anatomical changes caused by tooth loss, affecting the size and function of the upper airway space [23]. Utilizing self-reported questionnaires regarding sleep quality and daytime sleepiness (the Pittsburgh Sleep Quality Index, or PSQI, and the Epworth Sleepiness Scale, or ESS), Emami et al. [26] found no notable difference in sleep quality or perceived daytime sleepiness in edentulous elderly individuals who either wore or did not wear their dentures at night. In contrast, Mattia et al. [27] assessed sleep quality and the presence of sleep apnea in patients using mucosa-supported superior complete dentures and lower implant-retained complete dentures during the nighttime. Consequently, further research employing objective evaluations of sleep disorders within a controlled sleep laboratory setting is recommended to clarify this subject.

Additionally, the strongest indicators of musculoskeletal pain were found in individuals aged 30–39 who possessed high body mass indexes, belonged to a lower socio-economic status, had sedentary lifestyles, experienced nonrestorative sleep, suffered from daytime sleepiness, exhibited symptoms of anxiety and depression, and reported a decreased quality of life [28]. Available data suggest a possible correlation between poor sleep and chronic pain, potentially fostering a cyclical relationship wherein one condition exacerbates the other [29–31]. In a study conducted, participants with sleep disorders reported higher instances of gingival bleeding compared to those without sleep disorders, leading to the potential increase of periodontitis risk [32–34]. Other researchers have observed a correlation between the severity of periodontitis and the quantity of dental plaque in individuals with obstructive sleep apnea [35, 36]. While Nizam et al.‘s [37] study found no statistically significant differences in periodontal clinical measurements between patients with and without obstructive sleep apnea, similar results have been obtained. These findings could be attributed to the fact that sleep disorders can affect the immune system and incite systemic inflammation [32]. Salivary levels of IL-6 and IL-33, which regulate cell recruitment amid the transition from acute to chronic inflammation, have been observed to increase in patients contending with obstructive sleep apnea, independent of the severity of their periodontal disease. Furthermore, these concentrations may factor into the development of periodontal disease in these patients [37].

While our research provides valuable insights, it does come with its limitations. A notable restriction is the use of secondary data like the NHANES dataset. This approach limits the availability of certain essential variables. For a more detailed understanding of dental care practices’ impact, it would be beneficial to gather extra information. This could include the specific techniques used during oral care routines, as well as detection of the usage of mouthwash or fluoridated toothpaste among participants. Additionally, we combined various sleep disorder aspects, like sleeplessness, sleep apnea, duration, and quality, which may have limited the depth of our analysis. Our study focused on the overall impact of these factors on sleep, but we acknowledge the need for more detailed research on each aspect and their interactions. Another limitation related to our study design; it is cross-sectional, which precludes us from establishing a causative relationship between depression and oral health. Finally, we need to address the potential for recall bias due to the reliance on self-reported data, which could lead to underestimated results.

According to the data and the accompanying statistical analysis, there is a significant relationship between the presence of sleep disorders and the condition of gums and teeth, the occurrence of pain in the mouth, periodontal diseases, and negative or embarrassed feelings. However, no significant relationship was found between such disorders and having difficulties with work or school and the presence of bone loss. Further research is warranted to strength the evidence regarding the possible association between sleep disorders and oral health.

Our study provides initial insights into the connections between sleep disorders and oral health, boasting implications for the fields of dentistry and public health. Dental researchers should view sleep disorders as a potential risk factor for unfavorable oral health outcomes and also consider the necessity for accessible oral health information for those individuals with sleep disorders. In addition, health professionals should disseminate information about the increased risk of poor oral health among individuals with sleep disorders through various communication channels.

## Data Availability

All data has been presented in the manuscript.
